# FBX8 degrades GSTP1 through ubiquitination to suppress colorectal cancer progression

**DOI:** 10.1038/s41419-019-1588-z

**Published:** 2019-04-25

**Authors:** Wang FeiFei, Xu HongHai, Yan YongRong, Wu PingXiang, Wu JianHua, Zhu XiaoHui, Li JiaoYing, Sun JingBo, Zhou Kun, Ren XiaoLi, Qi Lu, Lan XiaoLiang, Cheng ZhiQiang, Tang Na, Liao WenTing, Ding YanQing, Liang Li

**Affiliations:** 1 Department of Pathology, Southern Medical University, 510515 Guangzhou, Guangdong Province P. R. China; 2 Department of Pathology, Nanfang Hospital, Southern Medical University, 510515 Guangzhou, Guangdong Province P. R. China; 3 Guangdong Province Key Laboratory of Molecular Tumor Pathology, 510515 Guangzhou, Guangdong Province P. R. China; 4 Department of General Surgery, The Third Affiliated Hospital, Southern Medical University, 510515 Guangzhou, Guangdong Province P. R. China; 5 Department of General Surgery, Nanfang Hospital, Southern Medical University, 510515 Guangzhou, Guangdong Province P. R. China; 6 Department of Pathology, Shenzhen People’s Hospital, 515020 Shenzhen, Guangdong Province P. R. China

**Keywords:** Colorectal cancer, Cell invasion

## Abstract

F-box only protein 8 (FBX8), as a critical component of the SKP1-CUL1-F-box (SCF) E3 ubiquitin ligases, has been associated with several malignancies through interacting with a member of proteins. However, the substrates of FBX8 for destruction in the progression of colorectal carcinoma (CRC) need to be explored. Here, we show that loss of FBX8 accelerates chemical-induced colon tumorigenesis. FBX8 directly targets GSTP1 for ubiquitin-mediated proteasome degradation in CRC. GSTP1 promotes the proliferation, invasion, and metastasis of CRC cells. Furthermore, GSTP1 is upregulated in CRC tissue samples and predicts poor prognosis of CRC patients. The inactivation of FBX8 negatively correlated with increased levels and stability of GSTP1 in clinical CRC tissues and FBX8 knockout transgenic mice. These findings identify a novel ubiquitination pathway as FBX8-GSTP1 axis that regulates the progression of CRC, which might be a potential prognostic biomarker for CRC patients.

## Introduction

Colorectal cancer (CRC) ranks fourth among all the malignancies worldwide^1^. Although the overall utilities of surgery, chemotherapy and radiotherapy control many localized tumors, they fail to restrict the development of tumor metastasis^2^. Therefore, it is imminently required for further elucidation of the molecular mechanisms underlying tumorigenesis and pathogenesis of CRC.

F-box proteins act as critical components of the SCF ubiquitin-protein ligase complex and primarily determine substrate specificity of ubiquitination through their direct interaction with substrates^3^. Dysregulation of F-box protein-mediated proteolysis often leads to human malignancies^4^. F-box only protein 8 (FBX8) contains an F-box domain and a putative Sec7 domain^5^. As reported, FBX8 has E3 ligase activity mediating the ubiquitination of the GTP-binding protein ARF6 and inhibits ARF6-mediated cell invasion activity in breast cancer^6^. Moreover, FBX8 is a novel c-Myc binding protein and c-Myc induces cell invasive activity through the inhibition of FBX8 effects on ARF6 function^7^. Downregulation of FBX8 correlates with tumor grade and poor prognosis in human glioma^8^. We have recently showed that FBX8 is lost in hepatocellular cancer, gastric cancer, CRC and correlated with poor survival in patients^9–11^. Moreover, FBX8 is a metastasis suppressor in CRC^10^. However, the substrates of FBX8 in the progression of CRC need to be further illustrated.

Glutathione S-transferases (GSTs) are phase II metabolizing enzymes and function in xenobiotic biotransformation^12^, drug metabolism, protection against oxidative stress^13–15^, modulating cell proliferation and signaling pathways^16^. The Pi class glutathione S-transferase P1 (GSTP1), as an isozyme of GST, is a major regulator of cell signaling in response to stress, hypoxia, growth factors, and other stimuli^16^. In addition, GSTP1 is over-expressed in a variety of human cancers, including bladder cancer^17^, ovarian cancer^18^. By contrast, downregulation of GSTP1 is observed in breast cancer^19^, hepatocellular cancer^20^, and prostate cancer^15^. But the function and regulatory mechanisms of GSTP1 in the progression of CRC remains unclear.

Here, we identify that FBX8 suppresses CRC progression by ubiquitin-dependent degradation of GSTP1. Moreover, upregulation of GSTP1 in CRC tissues is associated with poor prognosis of patients.

## Materials and methods

### Transgenic mice generation and treatments

FBX^+/–^, FLP^+/–^, and EIIa-Cre^+/–^ mice were brought from Shanghai Research Center for Model Organisms. All mice were on a C57BL/6 background and housed under standard pathogen free conditions. The mouse FBX8 gene contains six exons, spanning 16,186 kb of genomic DNA sequence (http://asia.ensembl.org/index.html?redirect=no). We isolated an FBX8 genomic DNA fragment containing all six exons from RPCI-22 129/SvEvTac mouse BAC library (Bacterial Artificial Chromosome, Source Bioscience Ltd.UK). One loxP sequence was inserted into each of the two EcoRV sites in the second and third introns, respectively. Then an frt-flanked neo expression cassette was inserted immediately to the neomycin sequence in intron 3 for positive selection^21^. After that, we employed heterozygous EIIa-Cre transgenic mice, which express Cre in early stage of embryogenesis thereby deleting the FBX8 from parenchymal and non-parenchymal colorectal cells^22^. Tail DNA was digested with FokІ (New England BioLabs) and genotyped by PCR and the primers were listed in Supplemental Table 1. As the recombination of loxP sequences occurs only in the presence of Tamoxifen, adult mice were treated with Tamoxifen (20 mg/kg, Sigma) for 5 days by single gavage to induce Cre recombinase in a broad range of tissues^23^. Azoxymethane (AOM) and Dextran sodium sulfate (DSS) were used to induce colorectal tumorigenesis in transgenic mice. Mice were injected with AOM via peritoneal cavity at 10 mg/kg per mouse and one week later they were treated with water contained 3% DSS for 5 days. Then they were rest for 1 week. The cycle was repeated three times for 6 months. All mice were euthanized at 25-weeks-old.

### Mass spectrometry

Each lane of the prepared SDS-PAGE gel was added with 10μl degeneration protein of HEK293T cells and then the gel was placed in a prepared coomassie brilliant blue dye with low velocity for 1 h at room temperature. Then the gel was eluted with destaining solution for 10 min at three times and scanned (Epson Expression 11000XL Scanner). The different expression protein sites of IgG and FBX8 in the gel were cut and placed into special EP tube. The results of the protein sites were analyzed by mass spectrometry.

### Co-immunoprecipitation (Co-IP)

In brief, the extracts of SW620 cells were blocked with IgG or protein A + G Agarose 2 h at 4 °C to get rid of unspecific protein binding and then they were incubated with anti-FBX8 or anti-GSTP1 overnight at 4 °C. The protein A/G-agarose was separated out by centrifugation at 4 °C, 2500 rpm. PVDF membranes were blocked with 5% skim milk for 1 h at room temperature and incubated with FBX8 (1:100) and GSTP1 (1:200) antibodies overnight at 4 °C at the dilutions listed below in 5% skim milk. Protein bands were visualized using enhanced chemiluminescence kit HRP (FD bio-femto ECL Kit).

### Glutathione S-transferase (GST) pull-down assay

The interaction of truncated FBX8 with GSTP1 was examined in HCT116 and SW620 cells by GST-mediated pull-down assays (Thermo Scientific, Rockford, IL). Recombinant GST-FBX8-1, GST-FBX-Sec7 proteins were expressed and purified. Purified GST-FBX8, GST-FBX-Sec7 fragments were bound to glutathione resin as a GST-fusion protein and incubated with GSTP1 at 4 °C for 2 h. After extensive washing with assay buffer, the complex was eluted with 5 mM reduced glutathione and the bound protein complexes were disrupted. Then, the proteins were separated on SDS-PAGE and western blotting.

### Immunoprecipitation

In brief, the vector containing cytomegalovirus (CMV) promoter driven pReceiver-M06-HA-ubiquitin was generated and then transfected into FBX8 overexpression SW480 cells and control cells. After 48 h, the extracts were prepared in lysis buffer. Lysates were precleared with protein A/G-sepharose beads (GE Healthcare) and then incubated with anti-HA overnight at 4 °C. After that, the beads were washed three times with lysis buffer and separated out by centrifugation at 4 °C, 2500 rpm. The precipitates were subjected to immunoblotting.

### Orthotopic mice metastatic models

For orthotropic metastasis assay, surgical orthotopic implantation of 3 × 10^6^ CRC cells were injected into the serosa of cecum was performed in 4–6-week-old male athymic BALB/c-nu/nu mice. The mice were euthanized 60 days after surgery. The cecum orthotopic tumors were detected by H&E staining and quantified by counting liver metastatic nodules.

## Results

### FBX8 deficiency promotes AOM/DSS-induced colon tumorigenesis

**Fig. 1:**
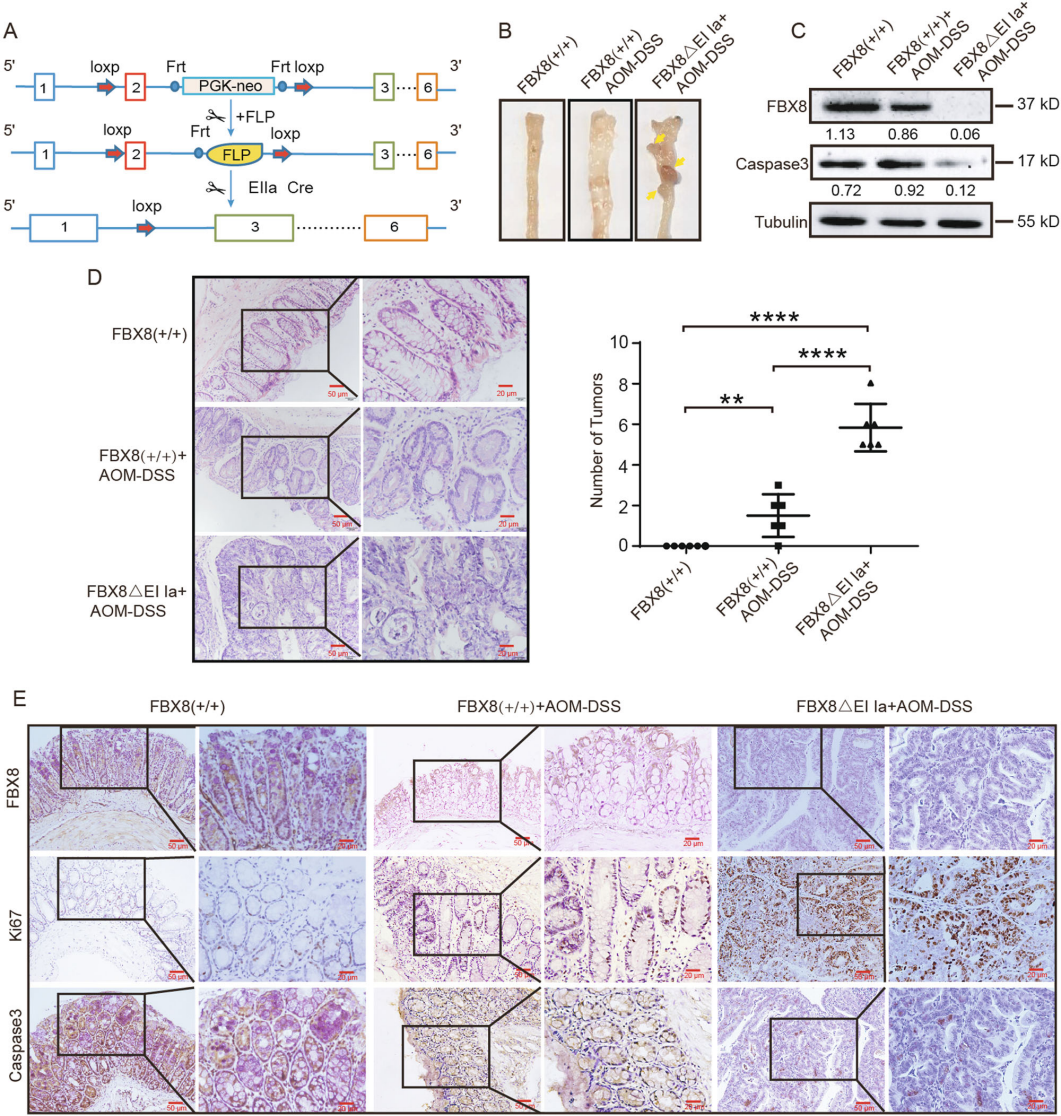
Generation of FBX8 Knockout Transgenic mice. **a** A diagram depicting the FBX8 conditional knockout (CKO) allele. FLP excised the Neo cassette, leaving behind a cko allele. Cre recombinase deletes the region between the two loxP sites. One loxP and one F3 site still remain after Cre-loxP-mediated deletion. **b** General pictures of colon in FBX8-WT or FBX8 KO mice treated with or not AOM-DSS. **c** The protein levels FBX8 and caspase 3 analyzed by western blotting in FBX8-WT or FBX8 KO mice treated with or not AOM-DSS. Tublin as a internal reference. **d** The colon tissue sections of FBX8-WT or FBX8 KO mice treated with or not AOM-DSS were stained with H&E. Scale bars: 50 or 20 μm. **e** The colon tissue sections of FBX8-WT or FBX8 KO mice treated with or not AOM-DSS were subjected to IHC staining using an antibody against FBX8, Ki-67, caspase 3. Scale bars: 50 or 20 μm. **P < 0.01, ****P < 0.0001

To explore the biological functions of FBX8 in vivo, we generated FBX8 knockout mice (Fig. 1a). The offspring were genotyped with tail DNA by PCR for the presence of the targeted FBX8 KO, FBX8 f/+, FBX8-WT, and Ella Cre (f/+) allele (Fig. S1A, B). Western blotting and IHC results validated the absence of FBX8 in colonic mucosal epithelia in FBX8 KO mice (Fig. S1C, D). No spontaneous colon tumors were observed in FBX8-KO mice. To address the role of FBX8 reduction in tumorigenesis of CRC, we used AOM and DSS protocol^24^. As expected, FBX8-KO mice generated obvious dysplastic epithelia and tumors after induction of AOM-DSS compared with WT littermates (Fig. 1b). Importantly, the FBX8-KO mice showed a marked increase in tumor number and size. Microscopically, in FBX8-KO/AOM-DSS group, we observed cancerous tissues infiltrating in the submucous layer in 3/6 mice, carcinoma in situ in 2/6 mice, and adenoma in 1/6 mice. However, in WT littermates, low-grade dysplasia in the mucosa were seen in 4/6 mice, high-grade dysplasia in 2/6 mice (*P* = 0.0057, *P* < 0.0001, *P* < 0.0001; Fig. 1d). Results of western blotting and IHC revealed that FBX8 was lost in FBX8-KO/AOM-DSS mice. Meanwhile, the level of Caspase 3 was decreased and the level of Ki-67 was increased in FBX8-KO/AOM-DSS mice, compared with FBX8-WT and FBX8-WT/AOM-DSS mice, which were consistent with the supression of FBX8 in cell proliferation of CRC^9^ (Fig. 1c, e). These data suggest that depletion of FBX8 enhances tumorigenesis in the murine AOM/DSS-induced colitis-associated CRC model.

### FBX8 controls the ubiquitylation and degradation of GSTP1 in CRC

**Fig. 2:**
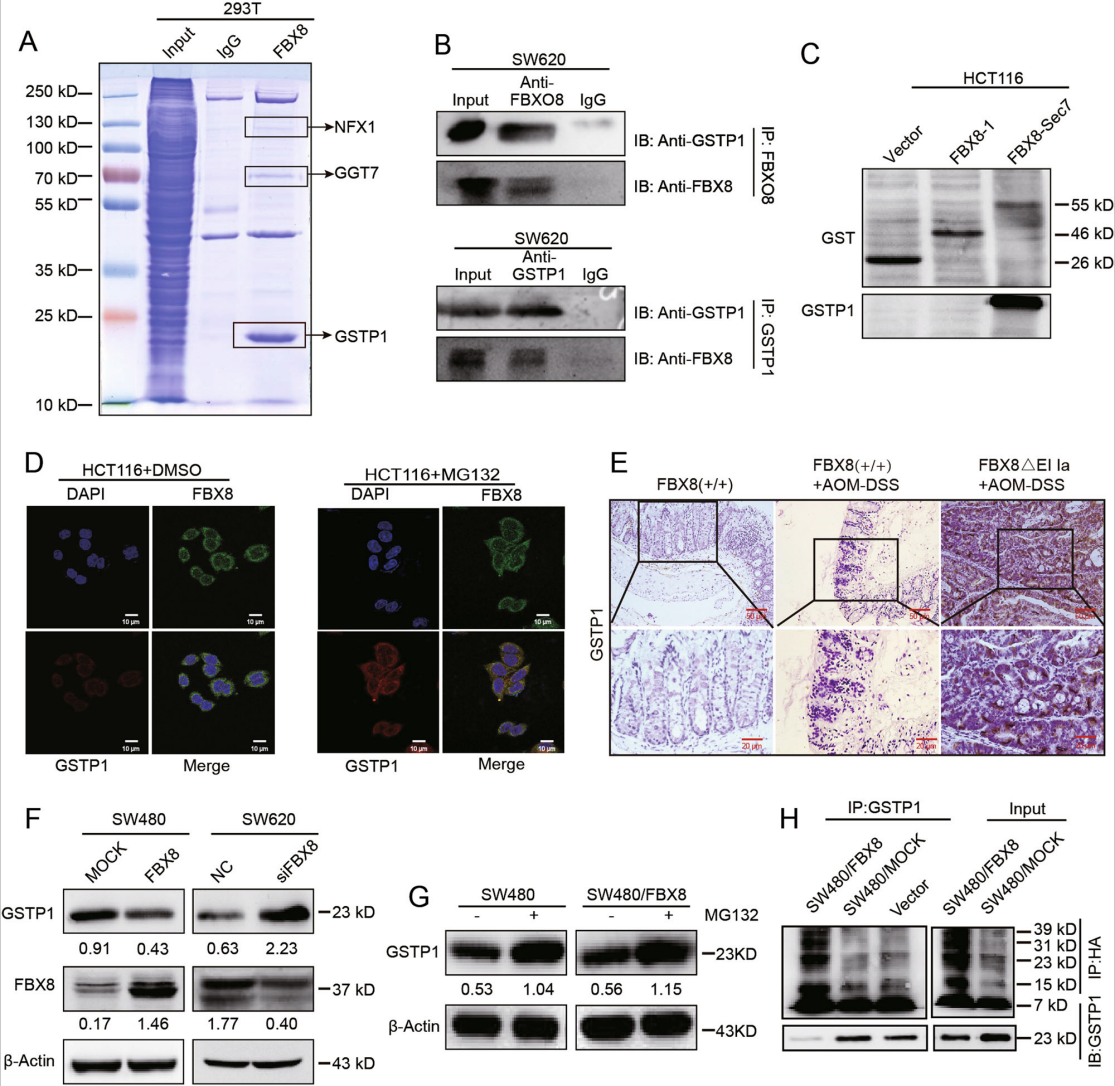
GSTP1 is the downstream ubiqitination target protein of FBX8. **a** Image of SDS-PAGE separation gel after staining by Coomassie brilliant blue. **b** FBX8 or GSTP1 were immunoprecipitated from SW620 cell lysates, analyzed by SDS-PAGE, and probed for FBX8 and GSTP1, respectively. **c** GST Pull-Down experiments of different domains of FBX8 protein with GSTP1. **d** HCT116 cells treated with DMSO or MG132 were seeded on class bottom cell culture dishs for 24 h, and stained for FBX8 (green), GSTP1 (red), and DAPI (nucleus). Scale bars represent 10 μm. **e** The expression of GSTP1 in colon tissue sections of FBX8-WT and FBX8 KO mice treated with or without AOM-DSS. Scale bars represent 50 or 20 μm. **f** SW480 were transfected with control or FBX8 vector or SW620 cells were transfected with control or FBX8 siRNA. Western blot analysis of FBX8 and GSTP1, β-actin as a internal reference. **g** SW480 were transfected with control or FBX8 vector, along with or not MG132. Western blot analysis of FBX8 and GSTP1, β-actin as a internal reference. **h** GSTP1 were immunoprecipitated from the lysates of SW480 cell treated with FBX8 vector or not, analyzed by SDS-PAGE, and probed for HA and GSTP1, respectively

F-box family mainly contributes to protein ubiquitination and thus regulates tumor progression^3^. To identify the potential degradation substrates of FBX8, we stably transfected HEK293T cells with FBX8 lentivirus (Fig. S2A) and then performed mass spectrometry analyses to screen downstream ubiqitinated proteins of FBX8. Results showed that NFX1, GGT7,or GSTP1 protein might directly combine with FBX8 (Fig. 2a). We chose GSTP1 as a potential substrate of FBX8 because of its highest score in peptide fragment blast (Fig. S2B). Co-IP analyses were performed to examine whether FBX8 physically interacted with GSTP1. The existence of GSTP1 was detected in the immunoprecipitates obtained with antibody against FBX8 in SW620 cells (Fig. 2b). GST pull-down assay further identified that GSTP1 directly bound to Sec7 domain of FBX8 (Fig. 2c). Immunofluorescence showed that positive signal of colocalization between GSTP1 and FBX8 was observed in the cytoplasm of HCT116 cells. The treatment of MG132 (a proteosome inhibitor) enhanced the colocalization of two proteins (Fig. 2d). After that, we examined whether FBX8 affected the level of GSTP1 in CRC tissues and cells. In the cancerous tissues of FBX8-KO/AOM-DSS mice, the expression of GSTP1 was obviously higher than in normal epithelial of FBX8-WT or dysplastic epithelial of FBX8-WT/AOM-DSS mice (Fig. 2e), and found that ectopic FBX8 in SW480 cells decreased the level of GSTP1, while depletion of FBX8 showed the opposite effect (Fig. 2f). Moreover, the treatment of MG132 (proteasome inhibitor) induced the increase of GSTP1 level in SW480/FBX8 cells (Fig. 2g). To test whether FBX8 degraded GSTP1 by the ubiquitin-mediated proteasome pathway, we introduced a vector containing HA-ubiquitin into SW480/FBX8 cells. Immunoprecipitation of GSTP1 followed by immunoblot analysis of HA showed that GSTP1 ubiquitination was obviously observed in control cells compared with SW480/FBX8 cells (Fig. 2h). The above data indicate that FBX8 induces ubiquitination degradation of GSTP1 in CRC.

### GSTP1 promotes proliferation, invasion, and metastasis of CRC cells

**Fig. 3:**
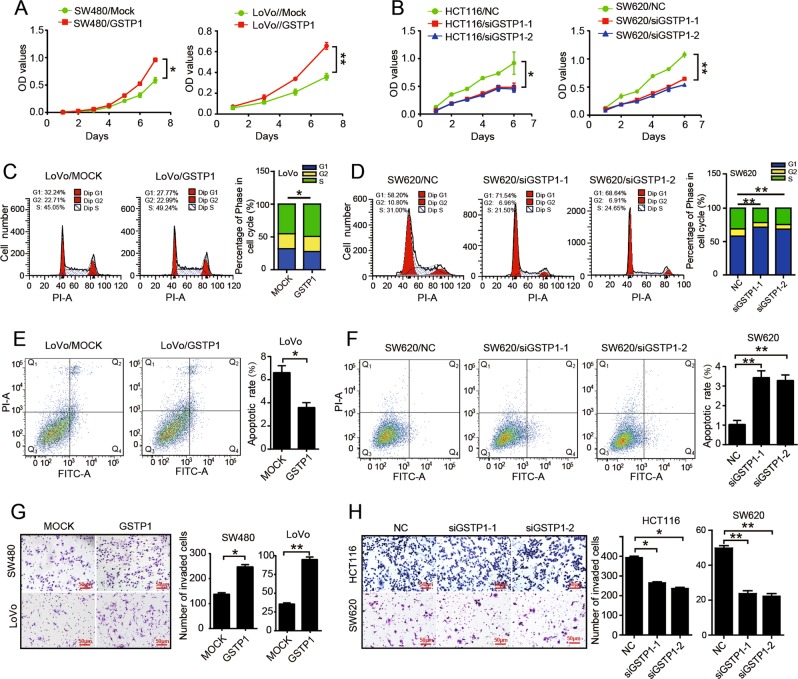
GSTP1 promotes proliferation and invasion in CRC cells. **a**, **b** Effect of GSTP1 on cell proliferation by CCK8 assays. **c**, **d** Effect of GSTP1 on cell cycle by Flow cytometry. **e**, **f** Effect of GSTP1 on cell apoptosis by Flow cytometry. SW480 and LoVo cells transfected with control or FBX8 vector. HCT116 and SW620 cells transfected with control or FBX8 siRNAs. Error bars represent mean ± SD from three independent experiments. **g**, **h** Effect of GSTP1 on invasion. Morphologic comparison of cells penetrating the artificial basement membrane was also shown. Scale bars represent 50 μm. *P < 0.05, **P < 0.01

Till now, the biological function of GSTP1 in the progression of CRC remains unknown. Thus, we examined the effect of GSTP1 on CRC cell behaviors in vitro and in vivo. According to endogenous expression of GSTP1 in six CRC cell lines (Fig. S3A), we chose LoVo, SW480 cell lines for overexpression of GSTP1, and SW620, HCT116 cell lines for GSTP1 knockdown. Results of western blot and RT-PCR validated high transfection efficiency (Fig. S3B, C). CCK8 assays showed that forced expression of GSTP1 caused a significant increase of the proliferation rate in SW480 and LoVo cells (*P* < 0.01, Fig. 3a), while GSTP1 depletion decreased the proliferative abilities in HCT116 and SW620 cells (*P* < 0.01, Fig. 3b). Cell cycle analyses showed that LoVo and SW480 cells treated with forced GSTP1 showed a significant decrease in the percentage of cells in the G1/G0 peak and an increase in the percentage of cells in the S and G2/M peak (*P* < 0.05, Fig. 3c, S3D). However, SW620 and HCT116 cells treated with GSTP1 siRNAs showed the opposite effect (*P* < 0.05, Fig. 3d, S3E). In addition, GSTP1 expressing LoVo and SW480 cells showed decreased rate of apoptosis (*P* < 0.05, Fig. 3e, S3A), while the rate of apoptosis was remarkably increased when GSTP1 was depleted in SW620 and HCT116 cells (*P* < 0.05, Fig. 3f, S4B). These results suggest that GSTP1 might promote CRC cell proliferation by eliciting cell cycle transition in S phase and prevented apoptosis.

We also evaluate the effect of GSTP1 on CRC invasion and metastasis. Results of in vitro invasion assay showed that overexpression of GSTP1 in SW480 and LoVo cells effectively strengthened the invasive abilities (*P* < 0.01, Fig. 3g), while suppression of GSTP1 in SW620 and HCT116 cells had the adverse effect (*P* < 0.01, Fig. 3h). In mice models of tumor growth and orthotopic metastasis established by subcutaneous and injected into serous of cecum with CRC cells, we found that GSTP1 enhanced primary tumor growth (*P* < 0.01, Fig. 5a) and significantly increased the number of metastatic liver nodules in mice (*P* < 0.05, Fig. 5b). These results provide evidence that GSTP1 is a positive regulator in the progression of CRC.

### GSTP1 is required for FBX8-induced CRC invasion and metastasis

**Fig. 4:**
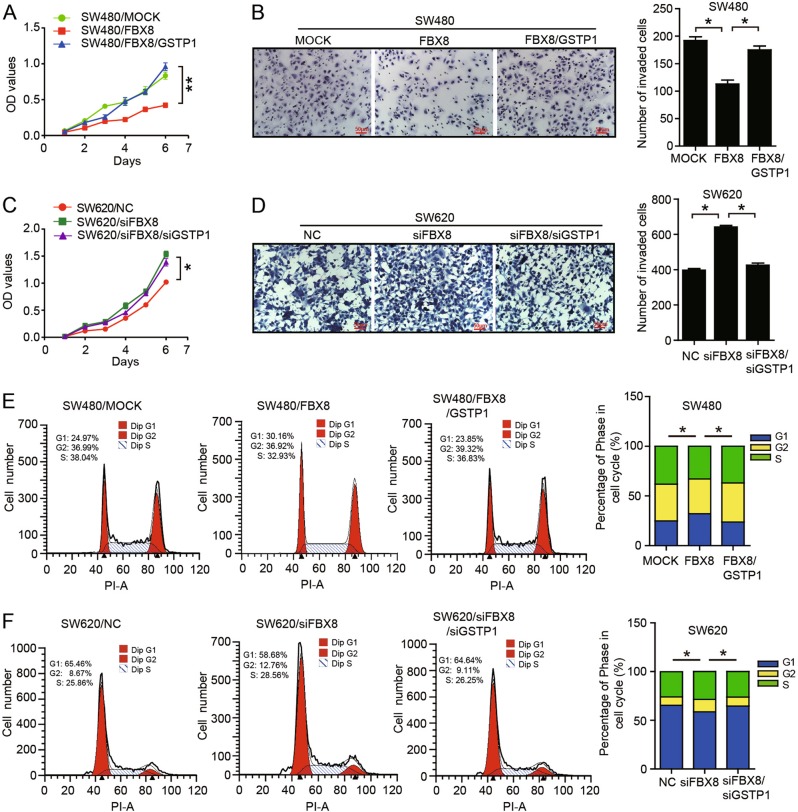
GSTP1 is necessary for FBX8-suppressived CRC proliferation and invasion. **a**, **b** Effect of cell proliferation and invasion in SW480 FBX8 overexpressing cells treated with or without GSTP1 by CCK8 assays or Boyden chamber. **c**, **d** Effect of cell proliferation and invasion in SW620 cells treated with FBX8 siRNA, along with or not GSTP1 by CCK8 assays or Boyden chamber. Morphologic comparison of cells penetrating the artificial basement membrane was also shown. Scale bars represent 50 μm. **e**, **f** Effect of cell cycle in SW620 cells treated with FBX8 siRNA, along with or not GSTP1 by Flow cytometry. *P < 0.05, **P < 0.01

**Fig. 5:**
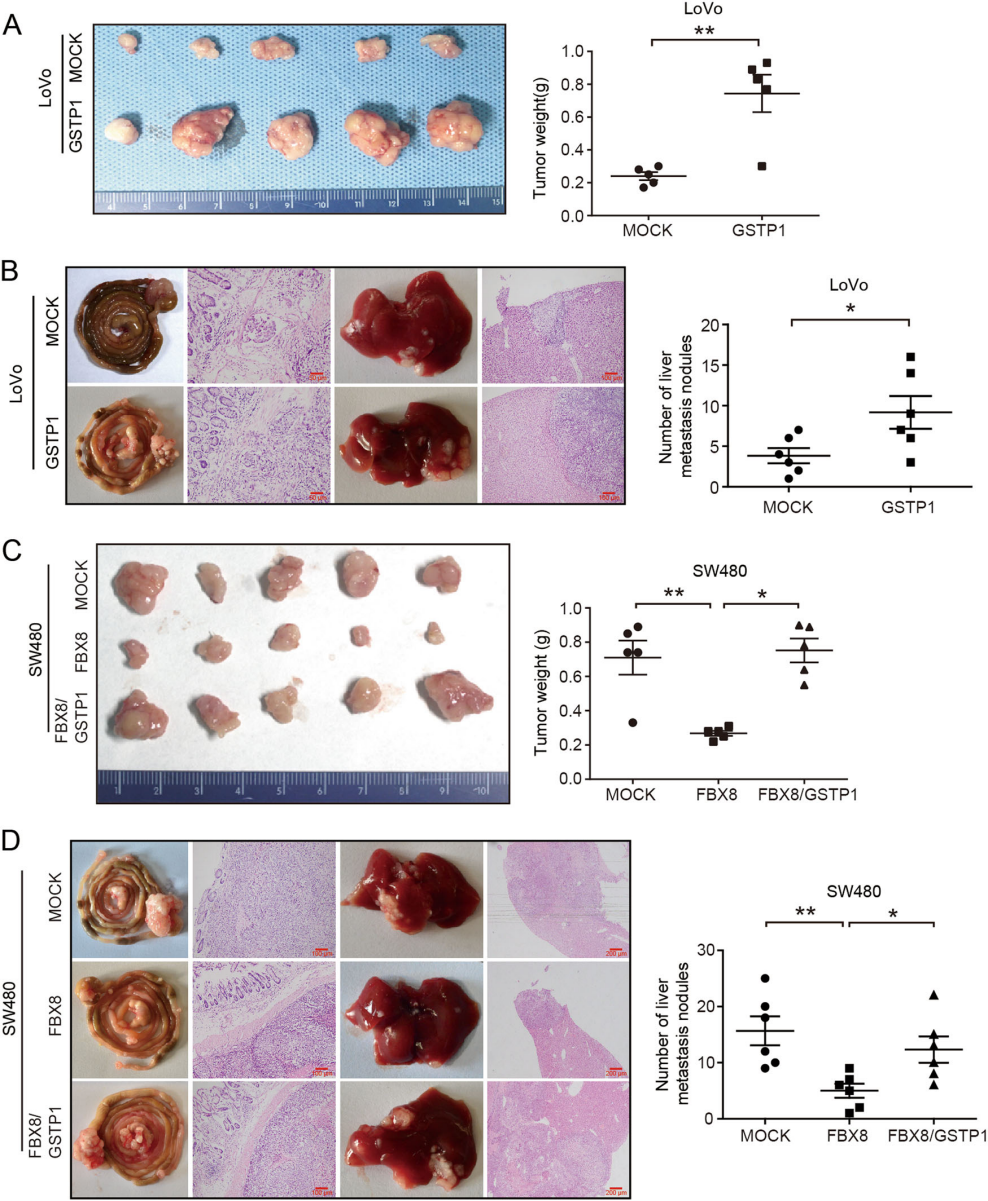
GSTP1 is necessary for FBX8-suppressived CRC proliferation and metastasis in vivo. **a** Subcutaneous tumors of mice injected with stable LoVo/MOCK and LoVo/GSTP1 cells. Quantification of tumor weight (*n* = 5). **b** Effects of LoVo/MOCK and LoVo/GSTP1 cells on liver metastases in vivo. The tumor sections were stained with H&E. Scale bars represent 50 or 100 μm. Quantification of metastatic nodules in individual mice (*n* = 6). **c** Subcutaneous tumors of mice injected with stable SW480/MOCK, SW480/FBX8, SW480/FBX8/GSTP1 cells. Quantification of tumor weight (*n* = 5). **d** Effects of SW480/MOCK, SW480/FBX8, SW480/FBX8/GSTP1 cells on liver metastases in vivo. The tumor sections were stained with H&E. Scale bars represent 100 or 200 μm. Quantification of metastatic nodules in individual mice (*n* = 6). Error bars represent mean ± SD from three independent experiments. *P < 0.05, **P < 0.01

To assess whether FBX8 suppresses CRC invasion and metastasis through ubiquitin degradation of GSTP1, we established stable cells with knockdown or overexpression of both FBX8 and GSTP1 (Fig. S4C). The rescue experiments showed that introduction of GSTP1 could alleviate the suppressions of FBX8 on the proliferation and invasion of SW480 cells (*P* < 0.01, Fig. 4a; *P* < 0.05, Fig. 4b), while silence of GSTP1 rescued the proliferation and invasion induced by FBX8 knockdown in SW620 cells (*P* < 0.05, Fig. 4c; *P* < 0.05, Fig. 4d). In addition, ectopic GSTP1 in SW480/FBX8 cells induced a significant decrease in the percentage of cells in the G1/G0 peak and an increase in the percentage of cells in the S and G2/M peak (*P* < 0.05, Fig. 4e). However, knockdown of GSTP1 in FBX8-depleting SW620 cells had the opposite effect (*P* < 0.05, Fig. 4f). In vivo tumorigenesis model, we found that overexpression of GSTP1 significantly rescued FBX8-induced suppression of tumor growth (*P* < 0.01, Fig. 5c). Moreover, FBX8 expressing cells produced less liver metastases compared with control cells, while GSTP1 could increased the number of metastatic liver nodules induced by FBX8 in vivo metastasis model (*P* < 0.01, Fig. 5d). These results demonstrate that FBX8 inhibits proliferation, invasion, and metastasis of CRC cells by downregulating GSTP1.

### GSTP1 is upregulated in CRC tissues and predicts poor survival of CRC patients

**Fig. 6:**
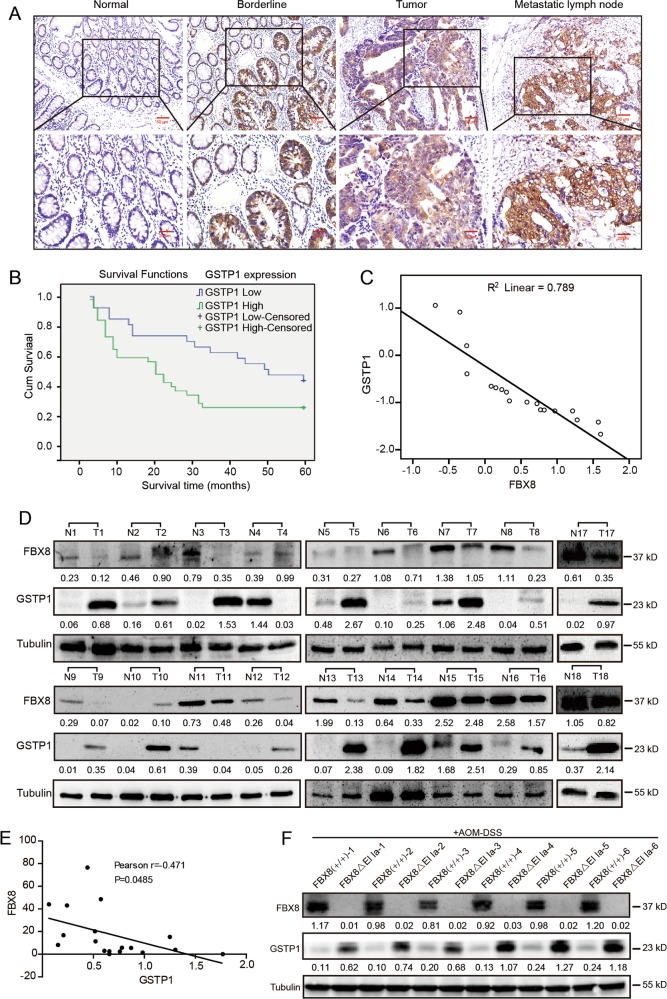
GSTP1 is up-regulated in colorectal carcinoma. **a** IHC staining of GSTP1 in normal tissues, CRC tissues and metastatic lymph node. Scale bars represent 50 or 20 μm. **b** Kaplane–Meier survival analysis of GSTP1 in CRC patients. **c** The relationship of expression between FBX8 and GSTP1 in 18 paired colorectal cancer tissue samples by RT-PCR (*r* = 0.789, *P* = 0.0018). **d** Western blotting was performed for GSTP1 and FBX8 in 18 paired fresh CRC tissues. **e** The relationship of expression between FBX8 and GSTP1 in 18 paired colorectal cancer tissue samples by western blotting (*r* = −0.471, *P* = 0.0485). **f** Western blotting was performed for GSTP1 and FBX8 in colon tissue of FBX8-WT and FBX8 KO mice treated with AOM-DSS

To assess the expression pattern and clinicopathological value of GSTP1 in CRC tissues, we detected the expression of GSTP1 in 136 cases of clinical paraffin-embedded CRC tissues by IHC. The expressions of GSTP1 were obviously higher in CRC tissues than in adjacent normal tissues (*P* < 0.001, Table S2, Fig. 6a). There was no significant difference of GSTP1 expression between primary tumor and metastatic lymph node. Also, no obvious difference of GSTP1 expression was shown between primary CRC tissues with metastasis and those without metastasis (Fig. 6a). Clinicopathological analyses showed that GSTP1 expression was correlated strongly with differentiation (*P* = 0.009), distant metastasis (*P* = 0.001), and lymphatic metastasis (*P* = 0.000, Table S3). The above results demonstrate that upregulation of GSTP1 may be associated with the malignant progression of CRC. We also discussed the correlation between GSTP1 expression and patients’ survival. By Kaplan–Meier curve assessment, patients with higher GSTP1 protein level had a significantly lower 5-year survival rate than those with low GSTP1 protein level (*P* = 0.007, Fig. 6b). From univariate analysis, GSTP1 expression (*P* = 0.018), differentiation (*P* = 0.000), and distance metastasis (*P* = 0.000) and lymphatic metastasis (*P* = 0.103) were significantly related to 5-year survival rate (Table S4). Multivariate analysis results showed that GSTP1 expression, differentiation, and distance metastasis might play a role in predicting the overall survival in CRC patients (*P* < 0.05, Table S4). These results show that GSTP1 expression is an independent prognostic marker for survival of CRC patients.

### GSTP1 expression is highly linked with FBX8 in CRC tissues

To evaluate the expression correlation of FBX8 and GSTP1 in CRC tissues, we detected the expressions of FBX8 and GSTP1 in 18 paired samples of fresh CRC tissues and fresh colon tissues of FBX8-WT and FBX8 KO mice treated with AOM-DSS. Results of RT-PCR and western blotting showed that GSTP1 was obviously upregulated in CRC and colon of FBX8 KO mice treated with AOM-DSS tissues, while FBX8 was downregulated in CRC tissues compared to adjacent normal tissues (Figs. 6d, f, S4D), and the level of FBX8 was negatively correlated with that of GSTP1 in clinical CRC tissues and cancerous tissues of FBX8-KO/AOM-DSS mice and FBX8(+/+)/AOM-DSS mice (Fig. 6c, *R*^2^ = 0.789, *P* = 0.000; Fig. 6e, *r* = −0.471, *P* = 0.0485). These data provide further support for direct and functional associations between FBX8 and GSTP1 in CRC tissues.

## Discussion

F-box proteins, as the substrate specifying subunit of SCF-type E3 ubiquitin ligases, are responsible for directing the ubiquitination of numerous proteins, which is essential for cellular function. Due to their ability to regulate the expression and activity of oncogenes and tumor suppressor genes, F-box proteins themselves play important roles in cancer development and progression^25^. FBX8 is a newly identified component of the F-box proteins family. In our previous study, we identified that it was a metastasis suppressor in CRC^10^. Here we successfully generated FBX8-KO transgenic model and found that FBX8 knockout promoted AOM/DSS-induced CRC oncogenesis. To identify the substrates of FBX8 is an important way to understand its funtion in tumors. Validated subatrates for FBX8 that have effects on cancer include mTOR^10^, Arf6^6^. In this study, we performed FBX8-IP and mass spectrometry analyses to screen potential ubiqitinated proteins of FBX8. We selected out GSTP1 as a candidate substrate of FBX8 and then validated that FBX8 could directly bind to GSTP1. Moreover, over-exppression of FBX8 promoted the ubiquitin degradation of GSTP1 and reduced its protein level in CRC cells. The treatment of MG132 in CRC cells prevented the degradation of GSTP1 induced by FBX8. These evidences firmly demonstrate that FBX8 directly targets GSTP1 for ubiquitin degradation in CRC.

GSTP1 is one of the major members of GST supergene family of phase II metabolic enzymes, which involves in detoxifying carcinogens of cellular defense system^26^. Due to the polymorphisms of GSTP1, its functions in tumors remain controversial. GSTP1 overexpression increases cell proliferation in head and neck squamous cell carcinoma^27^. On the contrary, GSTP1 arrests bladder cancer T24 cells in G0/G1 phase and upregulates p21 expression^17^. GSTP1 also suppresses JNK downstream signaling and apoptosis in brain tumor cells^28^. High methylation of GSTP1 might be associated with the stages of breast cancer^29^ or prostate cancer^30^. In addition, GSTP1 is associated with resistance of therapeutic drugs in tumors. Increased expression of GSTP1 inhibits radiosensitivity in HeLa cells^31^. GSTP1 increases breast cancer risk and aggressiveness but enhances response to cyclophosphamide chemotherapy in North China^32^. Downregulation of GSTP1 directly enhances platinum drug chemosensitivity in ovarian tumor cell lines^33^. However, the function of GSTP1 in the progression of CRC keeps elusive. Our study presented strong evidence that GSTP1 promoted CRC cell proliferation by eliciting cell cycle transition in S phase and preventing apoptosis. GSTP1 also enhanced invasion and metastasis of CRC cells in vivo and vitro. Hence, we demonstrate that GSTP1 is a positive regulator in CRC progression.

Then we examined whether GSTP1 is involved in FBX8-induced CRC progression. The rescue functional assays showed that GSTP1 abolished the suppressive effects of FBX8 on cell proliferation, invasion, and metastasis. Although some studies show that GSTP1 is abnormally expressed in colorectal tumor^34–36^, but a large cohort of IHC results still lack. Our IHC results showed that GSTP1 was over-expressed in CRC and related to the poor survival of CRC patients. Furthermore, upregulation of GSTP1 in CRC tumors was correlated with differentiation, distance metastasis, and lymphatic meastasis of CRC patients. These data indicate that GSTP1 is sufficient for FBX8-induced proliferation, invasion, and metastasis and an independent prognostic marker for survival of CRC patients.

Previous studies have proved that FBX8 is downregulated in several tumors including CRC^8,9,11^ and the expression of GSTP1 in tumors is very different^15,17–20^. But no evidence shows the relationship between FBX8 and GSTP1 in tumors. Our results showed the negative expression correlation between FBX8 and GSTP1 in clinical CRC tissues and FBX8-KO/AOM-DSS mice, further supporting a close link between them in CRC.

To conclude, our work reveals a molecular mechanism controlling GSTP1 stablity and proposes that FBX8 loss contributes to CRC progression through allowing the accumulation of GSTP1, which has a critical role in CRC progression and is a useful prognostic marker for CRC patients. Confirming the precise role played by GSTP1 in CRC progression will not only increase our understanding of the biology of CRC, but also provide a potential, novel therapeutic molecular target marker for clinical CRC patients.

## Supplementary information

Figure S1

Figure S2

Figure S3

Figure S4

supplementary files-Figures legend

supplementary files-Tables

Supplementary material and Methods
