# Supplementary figures and images for: FBX8 degrades GSTP1 through ubiquitination to suppress colorectal cancer progression
Source: Cell Death Dis. 2019 Apr 25;10(5):351. doi: 10.1038/s41419-019-1588-z (PMC6484082; doi:10.1038/s41419-019-1588-z)

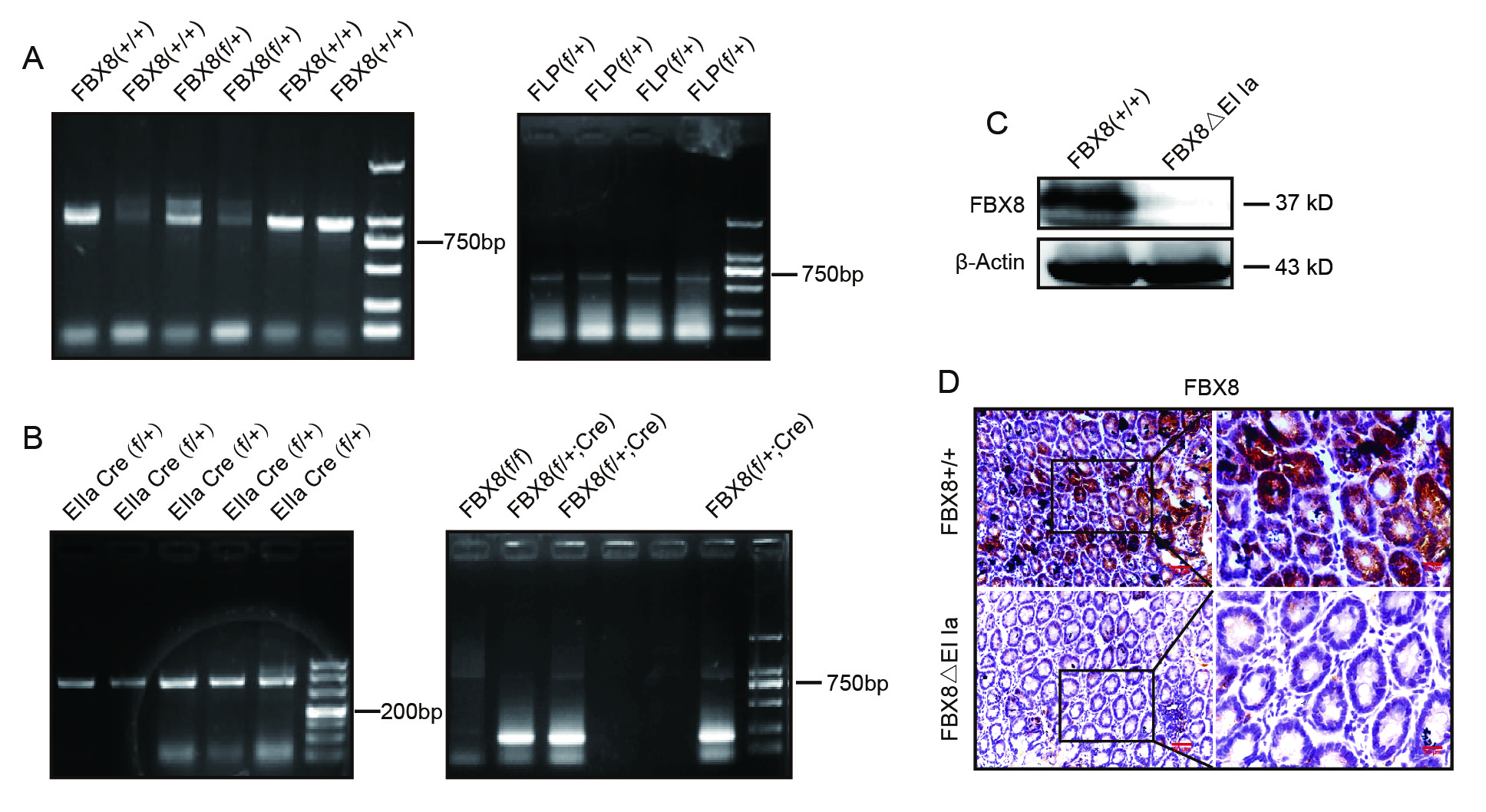

Supplement: Supplementary file 1 — Figure S1 [file 41419_2019_1588_MOESM1_ESM.jpg]

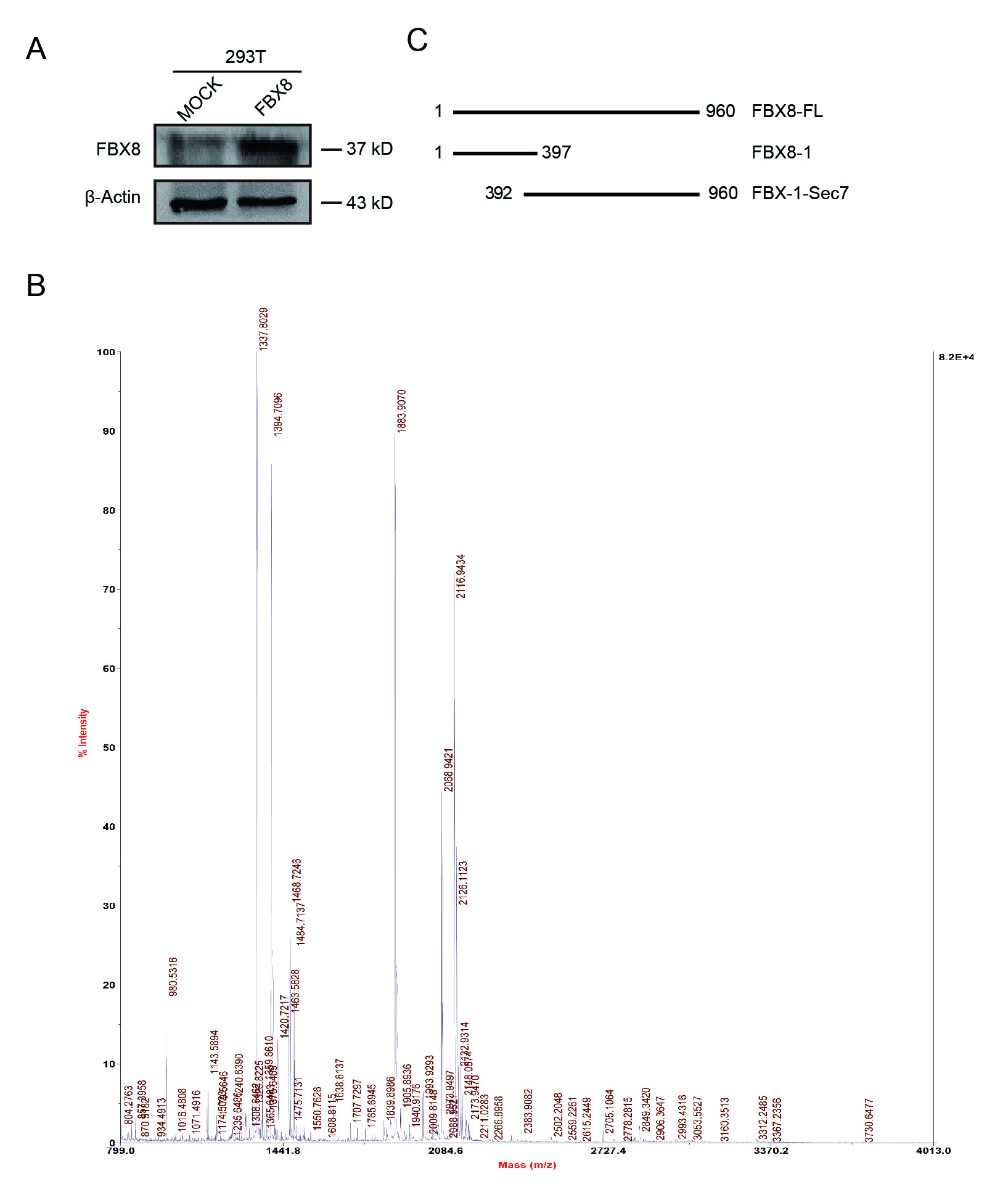

Supplement: Supplementary file 2 — Figure S2 [file 41419_2019_1588_MOESM2_ESM.jpg]

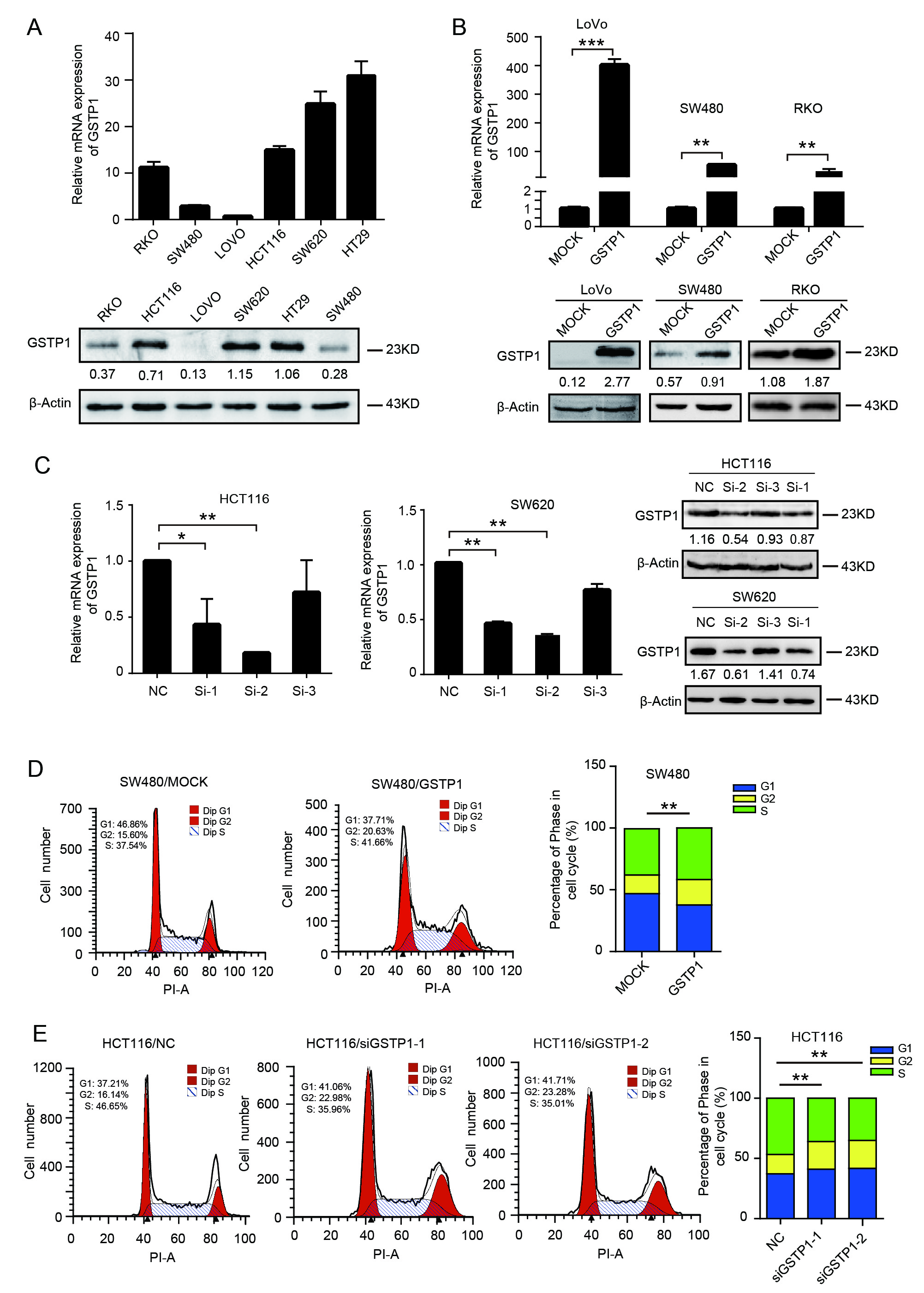

Supplement: Supplementary file 3 — Figure S3 [file 41419_2019_1588_MOESM3_ESM.jpg]

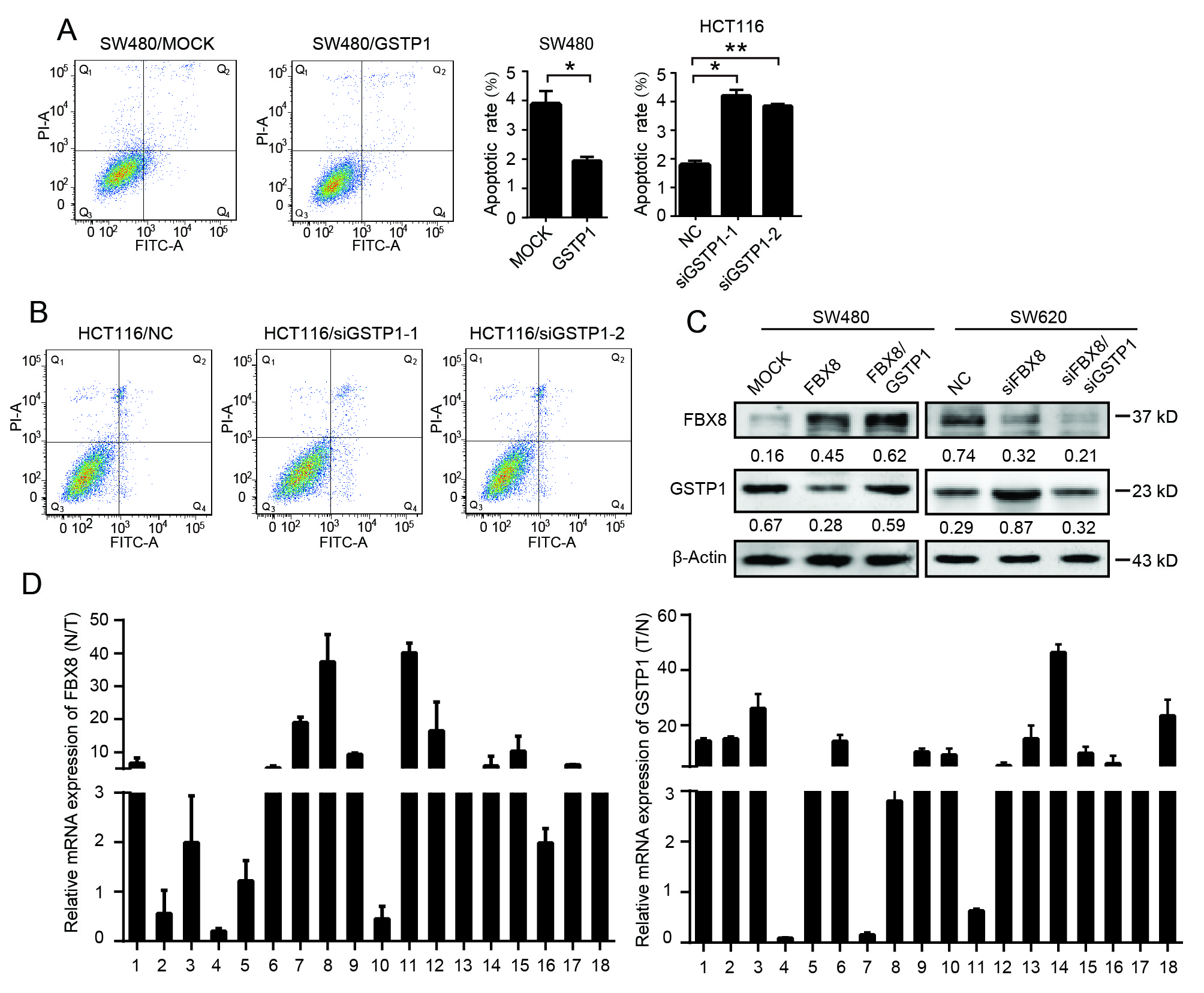

Supplement: Supplementary file 4 — Figure S4 [file 41419_2019_1588_MOESM4_ESM.jpg]
