# Supplementary material for: FBX8 degrades GSTP1 through ubiquitination to suppress colorectal cancer progression
Source: Cell Death Dis. 2019 Apr 25;10(5):351. doi: 10.1038/s41419-019-1588-z (PMC6484082; doi:10.1038/s41419-019-1588-z)
Supplement: Supplementary file 5 — supplementary files-Figures legend [file 41419_2019_1588_MOESM5_ESM.docx]

**Supplementary Figures**

**Figure S1 Identification of FBX8 knocked out mice.**

1. Representative PCR showing FBX8 from tail DNA of control (FBX8 -WT) and FBX8 f/+ mice. FBX8 -WT (0.95 kilobases, kb); FBX8 f/+ (0.95kb and 1.1kb). FLP f/+ mice, heterozygous (0.72kb). (B) EIIa Cre f/+ mice, heterozygous (0.35kb). Representative PCR showing FBX8 deletion from tail DNA of control (FBX8 -WT) and FBX8(f/+, Cre) mice. FBX8-WT, not deleted (0.95kilobases, kb); FBX8(f/+, Cre), deleted (0.95kb and 0.26kb). (C) The expression of FBX8 in colon tissues from FBX8-WT or FBX8 KO mice by western blotting. (D) The colon tissue sections of FBX8-WT or FBX8 KO mice were subjected to IHC staining using an antibody against FBX8.

**Figure S2 Selection of downstream ubiqitination target protein of FBX8.**

1. Efficiency of HEK293T cell lines transfected with FBX8 by western blotting. (B) The result of mass spectra spectrographic analysis of FBX8 IP assays. (C) Diagrams of the domains of FBX8.

**Figure S3 GSTP1 promotes proliferation and invasion in CRC cells**

1. The expression of GSTP1 in six CRC cell lines by western blotting and q-PCR.

(B) Efficiency of LoVo, SW480 and RKO cell lines transfected with GSTP1 by western blotting and q-PCR. (C) Efficiency of HCT116 and SW620 cell lines transfected with GSTP1 siRNAs by western blotting and q-PCR. (D-E) The effect of cell cycle in SW480 and HCT116 cells transfected with GSTP1 or GSTP1 siRNAs respectively.

**Figure S4 GSTP1 is up-regulated in colorectal carcinoma and highly linked with FBX8.**

(A-B) The effect of apoptosis by Flow cytometry in SW480 and HCT116 cells transfected with GSTP1 or GSTP1 siRNAs respectively. (C) The expressions of FBX8 and GSTP1 in SW620/MOCK, SW620/FBX8 or SW620/FBX8/GSTP1 cells or SW480/NC, SW480/siFBX8, SW480/siFBX8/siGSTP1 cells by western blotting. (D) Relative mRNA expressions of FBX8 and GSTP1 in 18 matched pairs of CRC samples.
