# Supplementary material for: FBX8 degrades GSTP1 through ubiquitination to suppress colorectal cancer progression
Source: Cell Death Dis. 2019 Apr 25;10(5):351. doi: 10.1038/s41419-019-1588-z (PMC6484082; doi:10.1038/s41419-019-1588-z)
Supplement: Supplementary file 6 — supplementary files-Tables [file 41419_2019_1588_MOESM6_ESM.docx]

**Supplementary Tables**

**Table S1 Primers to genotype FBX8 transgenic mice, FLP transgenic mice and EIIa Cre transgenic mice.**

| Gene | 5'to3' | 3'to5' |
| --- | --- | --- |
| FBX8 | GTCCTAGAATTCACTCAGAAATCCG | AGAATCCATATCATGGCTGTTTAGG |
| FLP | CACTGATATTGTAAGTAGTTTGC | CTAGTGCGAAGTAGTGATCAGG |
| EIIa Cre | ATTTGCCTGCATTACCGGTC | ATCAACGTTTTCTTTTCGG |

**Table S2 GSTP1 expression in CRC tissues and normal tissues among 136 cases of CRC patients.**

|  | High expression | Low expression |
| --- | --- | --- |
| Normal tissues | 21 | 115 |
| Tumor tissues | 82 | 54 |
| Pearson Chi-Square | 69.798 | |
| P-Value | <0.001 | |

**Table S3 Relationship between GSTP1expressions and clinicopathologic features of CRC patients.**

| **Features** | **Number of patients** | **High expression** | **Low expression** | **P** | **λ^2^** | |
| --- | --- | --- | --- | --- | --- | --- |
| **Age, y** |  |  |  | 0.098 | 2.734 | |
| <55 | 30 | 22 | 8 |  |  | |
| >=55 | 106 | 60 | 46 |  |  | |
| **Gender** |  |  |  | 0.09 | 2.879 | |
| Male | 92 | 60 | 32 |  |  | |
| Female | 44 | 22 | 22 |  |  | |
| **Differentiation** |  |  |  | 0.009 | 9.444 | |
| Well | 36 | 16 | 20 |  |  | |
| Moderate | 44 | 24 | 20 |  |  | |
| Poor | 56 | 42 | 14 |  |  | |
| **Distant metastasis** |  |  |  | 0.001 | 10.83 | |
| Y | 42 | 34 | 8 |  | | |
| ­N | 94 | 48 | 46 |  | | |
| **Lymphatic metastasis** |  |  |  | 0.000 | | 15.16 |
| **Y** | 112 | 76 | 36 |  | |  |
| N | 24 | 6 | 18 |  | |  |

**Table S4 Univariate and multivariate analysis of individual parameters for correlation with overall survival rate: Cox proportional hazard model.**

| **values** | **Univariate** | | **P-value** | **Multivariate** | | **P-value** |
| --- | --- | --- | --- | --- | --- | --- |
|  | **RR** | **CI (95%)** |  | **RR** | **CI (95%)** |  |
| **GSTP1** | 0.578 | 0.375-0.89 | 0.013* | 0.586 | 0.377-0.911 | 0.018* |
| **Age** | 1.299 | 0.801-2.107 | 0.288 |  |  |  |
| **Gender** | 0.884 | 0.564-1.386 | 0.592 |  |  |  |
| **Differentiation** | 0.528 | 0.402-0.695 | 0.000* | 0.51 | 0.381-0.681 | 0.000* |
| **Distance metastasis** | 2.917 | 1.884-4.516 | 0.000* | 2.868 | 1.815-4.531 | 0.000* |
| **Lymphatic metastasis** | 0.427 | 0.221-0.827 | 0.012* | 0.573 | 0.293-1.12 | 0.103 |

RR relative risk CI Confident interval *Statistically significant (P<0.05)
