# Supplementary material for: FBX8 degrades GSTP1 through ubiquitination to suppress colorectal cancer progression
Source: Cell Death Dis. 2019 Apr 25;10(5):351. doi: 10.1038/s41419-019-1588-z (PMC6484082; doi:10.1038/s41419-019-1588-z)
Supplement: Supplementary file 7 — Supplementary material and Methods [file 41419_2019_1588_MOESM7_ESM.doc]

**Supplemental Files-Materials and Methods**

**Cell lines, human tissue samples**

LoVo, SW620, HT29, SW480, HCT116, LS174T, RKO human CRC cell lines were purchased from American type culture collection (ATCC), Maryland, USA and human embryonic kidney 293T cells (HEK293T) were obtained from Shanghai Cell Bank of Type Culture Collection, Shanghai, China. CRC cell lines were cultured in Roswell Park Memorial Institute (RPMI-1640) (GIBCO, Gaithersburg, MD, USA) media and HEK293T cells were cultured in Dulbecco's Modified Eagle's medium (DMEM) (GIBCO, Gaithersburg, MD, USA) which is supplemented with 10% fetal bovine serum (GIBCO, Gaithersburg, MD, USA) maintained at 37℃ and 5% CO_2_. For inhibitors treatment, 5μM/L proteasome inhibitor MG132 (Cell Signaling, Boston, USA) was added in the cultured cells for 24 hours. Human fresh CRC tissues and Paraffin-embedded CRC samples were collected from 136 patients who underwent CRC resection without prior radiotherapy and chemotherapy at Department of General Surgery in Nanfang Hospital. Fresh CRC samples were collected immediately after resection, snap-frozen in liquid nitrogen, and stored at -80℃. All cases were followed up for 5 years to collected their complete clinical data. Approval for experiments on human tissues was obtained from Southern Medical University Ethics Committee for Human Genome Research.

**Plasmid construction and transfection**

Lentiviral vectors expressing FBX8 (pEZX-MR01 vector, GeneCopier, Shanghai, China) or GSTP1 (pEGFP-C1 plasmid vector, GeneCopier, Shanghai, China) were packaged using the pPACKH1 lentivector Packaging Kit (System Biosciences, California, USA) and used to infect CRC cells to obtain stable cells. Two truncated fragments of FBX8 were constructed as previously described [2].

**Proliferation, Cell invasion assays in vitro**

The proliferation and cell invasion assay of transfected CRC cells were determined as previously described [1, 2].

**Animal models in vivo**

In vivo tumor growth assay for four-to-six-week-old male athymic BALB/c-nu/nu mice, xenograft tumors were generated by subcutaneous injection of 3×10^6^ CRC cells, mice were euthanized after 21 days, tumors were measured with electronic balance.

**Western blotting**

Cellular protein expression was analysed via western blotting as previously described [2]. The western blotting of cell lysates was carried out for assessing protein expressions in RIPA buffer in the presence of mouse or rabbit antibodies to β-actin (Proteintech, Bioleaf Science, China, 1:1000), FBX8 (Abcam, London, England 1:100), GSTP1 (Abcam, London, England, 1:200), caspase3 (Abcam, London, England 1:400), HA (Proteintech, Bioleaf Science, China 1:500), GST (Abcam, London, England 1:1000).

**Real-time RT-PCR**

Cultured cells and the tissue samples RNA was extracted using Trizol reagent (Invitrogen, USA). The expression level of GSTP1 was analyzed by ABI PRISM 7500 Fast Real-Time PCR System. The relative mRNA levels were calculated using the comparative Ct method (ΔΔCt). Primer sequences for qRT-PCR are as follow:

GSTP1: Fwd: CGG GGT ACC ATG CCG CCC TAC ACC GT; Rev: CCG CTC GAG TCA CTG TTT CCC GTT GCC ATT; FBX8: Fwd: GAT TCG CCA AAG GAA ATA GC; Rev: GTT ACA AGG TCA TCC AAG AC.

**Immunofluorescence analyses**

For immunofluorescence of cells seeded at a density of 0.5 × 10^4^ cells on Confocal NEST dish glass bottom Petri dishes. After 24 hours, cells were fixed in 4% paraformaldehyde, permeabilized with 0.02%Triton-X/1x PBS, and blocked in 1x PBS + 10% fetal bovine serum and 1% BSA. Primary FBX8 (1:100), GSTP1(1:200) antibodies were incubated overnight at 4℃ at the dilutions listed below in 1x PBS. Secondary antibodies coupled to Alexa Fluor 488, or 594 (Invitrogen) were incubated 2 hr at room temperature. Nuclear DNA was stained with 4′, 6-diamidino-2-phenylindole (DAPI). Confocal images were taken by Olympus inverted fluorescence microscope and were outputted by PV10-ASW 1.7 viewer software.

**Immunohistochemistry (IHC)**

The sections of Paraffin-embedded CRC samples were deparaffinized and rehydrated, and retrieved by citric acid buffer(PH6.0) microwave 15 min antigen retrieval, endogenous peroxidase was inhibited with 0.3% H_2_O_2_ for 10 mins. Blocked in the 5% normal goat serum, primary GSTP1 (1:800) antibodies were incubated overnight at 4℃ at the dilutions listed below in 1x PBS. After secondary antibodies coupled, the visualization signal was developed with DAB. The stained tissue sections were scored as previously described [2].

**Statistical analysis**

For experiments among/between sample groups or three comparisons were analyzed by one-way ANOVA or independent samples T-test. Pearson’s correlation coefficient was used to measure the degree of the linear relationship of gene expression. P < 0.05 was considered significant. Error bars represent the mean ± SD.

Other experiments and materials were shown in supplemental data.

**References:**

1. Liang L, Li X, Zhang X, et al. MicroRNA-137, an HMGA1 target, suppresses colorectal cancer cell invasion and metastasis in mice by directly targeting FMNL2. Gastroenterology 2013; 144: 624-635.

2. Wang F, Zhang X, Yan Y, et al. FBX8 is a metastasis suppressor downstream of miR-223 and targeting mTOR for degradation in colorectal carcinoma. Cancer Lett. 2017 Mar 1;388:85-95.
